# Optimization of Environment-Friendly and Sustainable Polylactic Acid (PLA)-Constructed Triply Periodic Minimal Surface (TPMS)-Based Gyroid Structures

**DOI:** 10.3390/polym16081175

**Published:** 2024-04-22

**Authors:** Syed Saarim Razi, Salman Pervaiz, Rahmat Agung Susantyoko, Mozah Alyammahi

**Affiliations:** 1 Department of Mechanical and Industrial Engineering, Rochester Institute of Technology, Dubai Campus, Dubai P.O. Box 341055, United Arab Emirates; ssr4611@g.rit.edu (S.S.R.); msa1013@g.rit.edu (M.A.); 2 DEWA R&D Center, Dubai Electricity and Water Authority, Dubai P.O. Box 564, United Arab Emirates; rahmat.susantyoko@dewa.gov.ae

**Keywords:** 3D printing, additive manufacturing, fused deposition modeling, PLA, analysis, grey relational analysis

## Abstract

The demand for robust yet lightweight materials has exponentially increased in several engineering applications. Additive manufacturing and 3D printing technology have the ability to meet this demand at a fraction of the cost compared with traditional manufacturing techniques. By using the fused deposition modeling (FDM) or fused filament fabrication (FFF) technique, objects can be 3D-printed with complex designs and patterns using cost-effective, biodegradable, and sustainable thermoplastic polymer filaments such as polylactic acid (PLA). This study aims to provide results to guide users in selecting the optimal printing and testing parameters for additively manufactured/3D-printed components. This study was designed using the Taguchi method and grey relational analysis. Compressive test results on nine similarly patterned samples suggest that cuboid gyroid-structured samples perform the best under compression and retain more mechanical strength than the other tested triply periodic minimal surface (TPMS) structures. A printing speed of 40 mm/s, relative density of 60%, and cell size of 3.17 mm were the best choice of input parameters within the tested ranges to provide the optimal performance of a sample that experiences greater force or energy to compress until failure. The ninth experiment on the above-mentioned conditions improved the yield strength by 16.9%, the compression modulus by 34.8%, and energy absorption by 29.5% when compared with the second-best performance, which was obtained in the third experiment.

## 1. Introduction

Advancements around the world have increased the need for high-performance materials at minimal cost that can be used for the same applications as conventional materials. Researchers have explored additive manufacturing as a new technique to complement the existing conventional process. Additive manufacturing (AM) is carried out by using the layer-by-layer technique. The main difference of AM is that, unlike the conventional process, AM can produce very complex or intricate geometries without additional manufacturing costs. Additive manufacturing is also known as 3D printing. Three-dimensional printing can be used for automotive, biomedical, and machinery products. 

Amaya-Amaya et al. [1] discussed the use of 3D printing to develop auxetic composites of polylactic acid (PLA) reinforced with keratin materials to impart acoustic properties. Subsequently, an electro-acoustic experiment was performed to study the coefficient of sound absorption from auxetic composites of PLA with keratin materials. The experimental results showed that the sound absorption capacity of auxetic composites was greater than that of ordinary (honeycomb) and auxetic PLA geometries without the addition of keratin. Moreover, adding keratin helped reduce free spaces in cavities, which increased the airflow resistance and hence improved sound absorption. Also, increasing the interconnections between the cavities led to irregular transmission routes for sound waves, thus lowering the energy of the sound waves. Maran et al. [2] discussed the use of laser powder bed fusion to manufacture 3D auxetic structures. Using this process of manufacturing, the mechanical response of 3D auxetic structures was studied through key design parameters. These key design parameters included the vertical strut length, re-entrant strut length, strut thickness, and re-entrant angle. An experimental design approach with ANOVA statistical analysis methods assisted in the study of these key parameters. Further, it was found that Young’s moduli normal to and parallel to the vertical strut were heavily dependent on the length and thickness of the vertical strut. The yield strength was highly dependent on the length and thickness of the vertical strut as well. Next, the large deviations in Young’s moduli in the x- and y-directions were found to be 1.02 ± 0.07 GPa and 4.4 ± 0.1 GPa, respectively. In contrast, the yield strength in the x- and y-directions displayed minute anisotropy, which was found to be 45 ± 6 MPa and 45 ± 9 MPa, respectively. For Poisson’s ratio, there was no variation in the normal direction toward the vertical strut but a great deal of variation in the parallel direction toward the vertical strut.

Alomarah et al. [3] aimed to study the compressive properties of a novel 3D re-entrant chiral auxetic (RCA) structure. Regarding the topological features of both the re-entrant honeycomb and chiral honeycomb, the unit cell was developed with solid cubes and struts. The Multi Jet Fusion (MJF) 3D printing process was used to print six specimens from polyamide twelve. With the focus on a novel 3D RCA structure, uniaxial quasi-static compressive tests were applied in the x-, y-, and z-directions to analyze the Poisson’s ratio, energy absorption, and deformation of this 3D RCA structure. Next, ANSYS/LS-DYNA was used to develop finite element (FE) models, and then the corresponding experimental data were used to verify these models. Experimental microscopic measurements suggested that the MJF process is highly accurate, enough to produce sturdy parts with a smooth internal morphology. Also, the rotation function for the designed cubes was close to that of the cylinders of the 2D RCA structure. Plastic bending and buckling were the dominant deformation behaviors for samples compressed in the y-direction. For samples compressed in the x- and z-directions, plastic bending was by far the only dominant deformation behavior. Overall, the novel 3D auxetic structure under uniaxial compression along all three axes (x, y, z) facilitated the extreme shrinkage of the structure and consequently led to an enhanced load-carrying capacity. It was also determined that the RCA structure under a compressive load in the x-direction was more successful with regard to auxetic and energy absorption compared with the compressive loads along the y- and z-axes. The novel 3D RCA structure even displayed anisotropic properties. 

Wang et al. [4] discussed the viability of an interlocking assembly as one of the methods for fabricating 3D periodic auxetic cellular structures (PACSs). An interlocking assembly was used instead of additive manufacturing because 3D PACSs have complex geometries. With this method of fabrication, the mechanical properties, such as Poisson’s ratio and Young’s modulus with respect to the re-entrant angle, were studied with the help of uniaxial compression experiments and numerical simulations, which showed great qualitative and quantitative results. Furthermore, the results for strut thickness and the ratio of the vertical strut length to the oblique strut length were studied as well. As the structure became more re-entrant, the compression modulus increased. However, the highest value of Poisson’s ratio at a re-entrant angle of 45° was different from those in previous studies. As the struts thickened, the compression modulus of the structure rose, and Poisson’s ratio slowly moved from negative to positive and then reached Poisson’s ratio of the parent material at a moderate pace. In Nady et al.’s [5] work, mechanical and numerical homogenization models were developed to obtain an effectual elastic response with respect to large changes in the geometry of auxetic structures. Next, a strain nonlinear scheme was constructed to compute the stress–strain relationships of the repeated networks over a unit cell as a reference point. Homogenizations were utilized to study the effectivity of the nonlinear mechanical response of regular networks susceptible to planar and 3D auxetic behaviors. The pair stress portion of the homogenized constitutive code accounted for the influence of local rotations at the mesoscopic level and permitted the computation of bending response. This method was implemented on four planar auxetic repeated lattices and pyramid-shaped and 3D re-entrant lattices. The strain inflicted over the unit cell boundary caused the transition to auxetic behavior. As the stretch increased, the computed progression of Poisson’s ratio against the forced stretch resulted in an amplified auxetic response.

In the work by Lu et al. [6], two novel 3D cross-chiral structures exhibiting negative Poisson’s ratios were investigated. The first structure could be developed into the second by incorporating a star structure. Mathematical models for these two auxetic cellular structures were established using Timoshenko beam theory. The finite element method was employed to verify the results, obtaining Young’s modulus and Poisson’s ratio for the cellular structures in all principal directions. While the first structure exhibited anisotropic auxetic behavior, the second structure displayed constant auxetic behavior in all principal directions, with all Poisson’s ratios approaching the limit value of −1. Notably, for the same relative density, adding the star structure significantly increased the Young’s modulus of the second structure compared with the first. Gao et al. [7] proposed a new method for creating 3D structures with negative Poisson’s ratios using rotating rigid mechanisms as the starting point. This method led to a new class of 3D auxetic lattice structures. The authors then investigated the combined Young’s modulus and Poisson’s ratio in all principal axes, focusing on the elastic properties of a representative 3D auxetic lattice structure. Analytical predictions, experimental tests, and numerical simulations were employed in this investigation. The discussion also addressed the effects of structural geometrical variables and sample size on the elastic properties, particularly the nonlinear mechanical responses in the principal axes. The results suggested that the combined Poisson’s ratio of the 3D lattice structure in all principal axes could be tuned from positive to negative over a broader range compared with most traditional 3D auxetic structures. In another study, Gao et al. [8] explored the concept of highly stiff 3D auxetic lattice structures and a method for fabricating them using a high-performance continuous carbon fiber-reinforced polymer (CFRP) composite. Conceptual models were developed to predict the combined elastic properties of a representative structure in all principal axes, including shear moduli, Young’s moduli, and Poisson’s ratios. The directional dependence of these combined elastic properties was further investigated for these models. Numerical analysis and experiments were then used to scrutinize the compressive behavior of 3D auxetic lattice structures made with high-performance continuous CFRP. The results indicated that these 3D auxetic lattice structures were suitable for uniaxial loading, exhibit exceptional load-bearing capacity, and possess negative Poisson’s ratios.

In Wang et al.’s work [9], a mathematical model for a 3D re-entrant auxetic cellular structure was established using the energy method. The model considers the overlapping and axial extension/compression of struts, especially when they are thick, to ensure their applicability. Mathematical solutions for Poisson’s ratio and modulus in all principal directions were derived. To validate the model, numerical calculations with brick elements were performed on unit cell models with periodic boundary conditions. The results were then compared with existing mathematical formulae and experimental data. This comparison showed that the bending of the struts played a crucial role in deforming the structure when they were slender enough, while other mechanisms could be neglected. Conversely, when the struts became thicker (sturdy), mechanisms like shearing, bending, and axial loading became significant. Additionally, for these thicker struts, axial extension or compression could even play a crucial role in the lateral Poisson’s ratio. Yang et al. [10] investigated the mechanical properties of 3D re-entrant honeycomb auxetic structures fabricated using additive manufacturing. They developed a mathematical model based on the Timoshenko beam model and the large deflection beam model. Similar to Wang et al., they derived mathematical solutions for Poisson’s ratio, yield strength, and modulus in all principal directions, demonstrating a wide range of control over mechanical properties through geometric design. The model results were then compared with experimental data and finite element analysis. This verification confirmed the accuracy of the model in predicting the behavior of auxetic cellular structures, provided appropriate manufacturing elements were incorporated. However, the model’s accuracy diminished when higher-order coupling effects, such as warp locking, became significant under conditions of lower structural symmetry.

Ge et al. [11] investigated a novel 3D auxetic textile structure using finite element analysis. This structure incorporates the following three yarn systems: weft, warp, and stitch. Unlike traditional 3D textiles, this three-yarn system exhibited auxetic behavior (negative Poisson’s ratio) under compression, making it suitable for reinforcing auxetic composites. The study outlined the geometry of the structure and used a computational tool to develop a 3D finite element model. This model was then verified with experimental results. The authors exemplified the deformation process of the structure under different compression strains. The verified model successfully simulated the auxetic behavior for structures with varying yarn properties and structural parameters. They found that increasing the compression strain led to a more pronounced auxetic effect. Overall, the study demonstrated that yarn properties and structural variables significantly impact the auxetic behavior of the structure. Liu et al. [12] explored the design of 3D auxetic structures that possessed unique mechanical properties. They designed a structure with 2D draft angles to achieve adjustable out-of-plane buckling behavior (collapsing inwards). This behavior was achieved by manipulating the stiffness across the structure’s thickness. Using the finite element method, the study investigated the influence of radii and draft angles on the buckling behavior of these structures. The authors established key relationships among stress, strain, draft angle, and radius. These relationships helped explain the working principle behind the mechanical implementation of draft angle auxetic structures. Additionally, they modeled the buckling behavior using a laminate structure and verified the analytical results with experimental data.

Yu et al. [13] described a new 3D auxetic cellular structure with a unique negative Poisson’s ratio in both tension and compression. This structure was made by combining flat and wavy units into a frame. They studied four variations of this structure and found that the choice of units determines the response to tension and compression (positive or negative Poisson’s ratio in three directions). Experiments and simulations confirmed that these structures behave differently under compression and tension. They also considered material nonlinearities and showed that Poisson’s ratio can change from negative to positive for some models under increasing loads. This allowed for predicting the auxetic response based on the chosen units. Overall, this design offered the possibility of achieving zero, negative, or positive Poisson’s ratio. Choudhry et al. [14] investigated the energy absorption properties of redesigned re-entrant auxetic honeycombs created using 3D printing with geometric optimization. They studied how variations in strut length and joint angles affect the stiffness, strength, and energy absorption of these structures. Finite element simulations were compared with experimental data to validate the results. They analyzed the energy absorption behavior, stress–strain response, and deformation mode of the optimal structure compared to a traditional re-entrant auxetic honeycomb. The redesigned structure showed a 36% improvement in energy absorption capacity because of its increased failure strain and the presence of additional nodes with low rotational stiffness.

Lvov et al. [15] investigated re-entrant honeycomb auxetic structures using a combination of computer simulations, theoretical calculations, and experimental tests to determine Poisson’s ratio. They found ideal cell parameters for 3D-printed specimens made from thermoplastic polyurethane. These auxetic structures exhibited significantly higher fatigue resistance compared with their non-auxetic counterparts. After 500 compression cycles, the 3D-printed structures showed no signs of failure or delamination. Wollner et al. [16] focused on the impact of fluid and solid properties on the auxetic behavior of porous materials. Auxetic structures, defined by a negative Poisson’s ratio, widen when stretched and shrink when compressed. Their study proposed a novel auxetic model for porous rocks based on rotating rigid bodies. The model was then modified to incorporate the cracking behavior of intersecting elliptical cylinders. A 3D-printed model was created to evaluate its Poisson’s ratio. Finally, the authors numerically investigated the influence of the solid’s Poisson’s ratio and the fluid’s compressibility within the pore space on the overall auxetic behavior. Their findings suggested that a more compliant fluid and a solid with a lower Poisson’s ratio would result in a porous material with a lower Poisson’s ratio. Quan et al. [17] fabricated continuous fiber-reinforced thermoplastic composite (CFRTPC) auxetic honeycomb structures using a 3D printer with a planned printing path. This differed from typical auxetic honeycombs made from pure polylactic acid (PLA). The researchers tested the CFRTPC structures under in-plane compression and used scanning electron microscopy (SEM) to examine the resulting damage. They then employed a finite element (FE) printing path to simulate small and large deformations of the honeycombs. Additionally, they used an established mathematical model to predict the effective stiffness and Poisson’s ratio. The experimental measurements, FE results, and mathematical predictions were in good agreement. A structured parametric study was then conducted to identify the factors affecting the in-plane mechanical properties based on geometric variables. Unlike PLA structures, the continuous fibers in CFRTPC honeycombs prevented crack propagation within each ligament. Although adding these fibers increased the total mass by only 6%, it significantly improved the compressive stiffness and energy absorption by 86.3% and 100%, respectively. Furthermore, it resulted in lower Poisson’s ratios.

Shokri et al. [18] investigated separating the complex microstructure of auxetic materials into simpler structures with different deformation mechanisms. They focused on re-entrant and chiral structures and adapted a 2D re-entrant design for a 3D auxetic structure. This choice was made because re-entrant structures exhibit key auxetic properties. The researchers used numerical and energy methods from solid mechanics to analyze these properties. Understanding re-entrant structures provides a foundation for comprehending auxetic materials in general, which can aid the development of new material classes. Another study by Lvov et al. [19] explored the use of auxetic structures in products subjected to repeated stress (cyclic loads). They proposed a 3D auxetic structure with a Poisson’s ratio of 0.45. To investigate its mechanical properties, they created two physical test specimens as follows: one using 3D printing with Selective Laser Melting (SLM) and another through computer simulations (FEA). Finally, they compared the properties of this auxetic structure to a non-auxetic honeycomb structure. Cyclic testing revealed that the auxetic structure deformed uniformly, withstanding a maximum load of 12 kN before failure. In contrast, the non-auxetic structure lost stability under a lower load of 8 kN. Shen et al. [20] used electron beam melting (EBM) to create a novel 3D re-entrant lattice structure made of Ti-6Al-4V. This structure was achieved by merging 2D structural parts using new connections and topological methods. These 2D parts exhibited load-bearing capabilities and negative Poisson’s ratios under uniaxial loading. The study then involved the fabrication and testing of four different configurations under uniaxial compression. The deformation mechanism under compression was investigated, followed by applying beam theory to establish the relationship between mechanical properties and geometric design variables. Finally, finite element simulations of the compression test were conducted. The results showed good agreement among experimental data, simulations, and theoretical predictions. This new 3D structure surpassed traditional re-entrant lattice structures by offering superior mechanical properties, a wider design space, and a greater capacity for energy absorption.

Wang et al. [21] used beam-based crushing simulations to study the dynamic behavior of 3D re-entrant auxetic cellular structures. They investigated the complex deformation process under different crushing velocities and identified three distinct crushing modes based on relative density and velocity. Their findings showed that increasing both crushing velocity and relative density led to a higher crushing strength for the structure. Furthermore, they developed a mathematical formula for dynamic plateau stress that accurately reflected the simulation results. By analyzing this model, they explored the relationship between relative density and the energy absorption capacity of the structure. The results indicated that while increasing relative density increased the total plastic energy absorbed, the normalized plastic energy absorption per unit volume showed an opposite trend at velocities exceeding a critical value. Tino et al. [22] investigated the use of triply periodic minimal surfaces, also known as gyroid structures, for applications in radiotherapy phantoms. Their research focused on understanding the mathematical properties of gyroids, their impact on Hounsfield units (HUs), and fabrication methods. They employed techniques like optical microscopy, micro-computed tomography (µCT), and material Hounsfield equivalence to evaluate the manufacturing process of gyroid phantoms. Their findings revealed that gyroid phantoms fabricated with varying standard deviations resulted in an average HU between −900 and −390. Importantly, unlike conventional infill structures like grids and slits, gyroid phantoms exhibited isotropic standard deviation and HU values regardless of scanning direction. Additionally, their study suggested that altering the structural parameters of gyroids holds potential for future research in tissue imaging applications.

**Figure 1:**
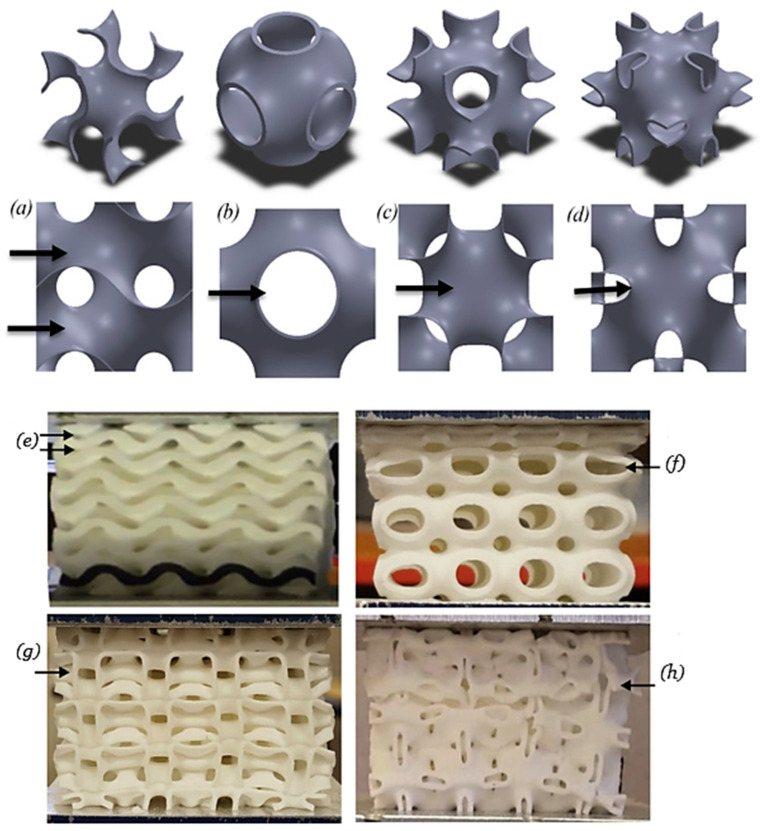
Deformation in TPMS cell structures under compressive testing: (**a**) gyroid, (**b**) primitive, (**c**) IWP, (**d**) Neovius, (**e**) deformed gyroid, (**f**) deformed primitive, (**g**) deformed IWP, and (**h**) deformed Neovius [adopted from 23 with kind permission from Elsevier].

Abueidda et al. [23] explored gyroid structures, a type of triply periodic minimal surface (TPMS), as shown in Figure 1. They investigate their mechanical properties using both computational modeling and experiments. First, 3D-printed gyroid samples of varying densities (made from PA 2200) were created. The Arruda–Boyce model was then implemented in finite element analysis. To ensure accuracy, the 3D-printed material properties were determined through tensile and compressive tests. This close link between experimental and computational results validates the approach. Compared with other TPMS structures, the gyroid structures exhibited unique mechanical properties. Holme et al. [24] aimed to replace polyurethane foams with 3D-printed gyroid structures to reduce pressure ulcers in patients. However, they first needed to understand how key features affect the mechanical response. Using fused filament fabrication (NinjaFlex, Fenner Precision Polymers, Manheim, PA, USA) and (Flexion X60 filaments, Flexion Extruder, ME, USA), they produced samples with six different unit cell geometries. These were then tested and compared to traditional polyurethane foams. Compression tests revealed that gyroid samples made from both filaments offered compressive responses comparable to conventional pressure ulcer foams. Notably, the solid volume fraction emerged as a critical geometric parameter for the compressive response of Gyroid structures. Zhu et al. [25] investigated 3D-printed tricalcium phosphate-bio glass scaffolds with a gyroid structure for bone defect treatment. Digital light processing (DLP) successfully manufactured the tricalcium phosphate/ bio glass composite (TCP/BG) scaffold with the desired gyroid structure. The researchers then analyzed the structural parameters, mechanical properties, and surface features of the TCP/BG gyroid scaffold. Compared with commercial bone grafts, their experiments showed the TCP/BG scaffold with a gyroid structure promoted bone ingrowth and integration while preserving the surrounding trabecular structure.

O. Al-Ketan et al. [26] focused on the mechanical properties of stochastic (randomly generated) sheet-based isotropic cellular materials. They used numerical and experimental methods to investigate these materials. The 3D design was based on a gyroid lattice, manufactured using the powder bed fusion (PBF) technique with 316L stainless steel powder at 15% relative density. Their proposed design approach involved creating single unit cells of TPMS-based structures and then rotating these patterned elements to generate stochastic structures. The results showed that periodic gyroid TPMS lattices exhibited better mechanical properties compared with the stochastic structures. Their study opened doors for further exploration of mathematically designed stochastic structures and their potential applications in engineering. Nejc Novak et al. [27] investigated the mechanical properties of two new hybrid TPMS cellular lattice designs (diamond and gyroid). These lattices combined longitudinal and radial elements of both gyroid and diamond structures. The specimens, fabricated using the PBF technique with stainless steel 316, were tested under dynamic and quasi-static loading conditions. The results showed that longitudinal hybrid lattices exhibited a distinct hardening behavior, while radial hybrid lattices displayed a more uniform and constant response. This difference was attributed to the way the radially spaced lattices deformed concurrently in the radial hybrid design compared with the longitudinal one.

Nejc Novak et al. [28] investigated how well different cellular structures made from stainless steel 316L with a TPMS sheet-based design withstood compressive forces. They compared the following four types: diamond, gyroid, IWP, and primitive. The structures were fabricated using a powder bed fusion technique and tested under various loading conditions. Their findings showed that the diamond lattice offered the highest strength and energy absorption. M. Khalil et al. [29] focused on heat transfer in heat sinks with mathematically designed TPMS lattices (diamond and gyroid). They used laser powder bed fusion to create these structures and analyzed their thermohydraulic performance. Their results showed that the gyroid sheet structure had the highest pressure drop but the lowest friction factor because of its large pores. Meinig et al. [30] investigated the thermal, mechanical, and morphological responses of injection-molded PLA with respect to processing parameters, namely, melt temperature, mold temperature, D isomer content mold cooling time, etc. Their study revealed that crystalline content increased by increasing the cooling time in the mold. Karimi et al. [31] investigated the cyclic compressive behavior of PLA. Their study utilized a numerical model to study the cyclic behavior of PLA. It was observed that elastic stiffness rose in the first cycle, but in later cycles, the amount of increase decreased. The default settings of the 3D printer, as seen in Table 1, were noted and were kept constant throughout the printing procedure.

Within the existing body of literature, there is a noticeable lack of knowledge about the optimization of input parameters, namely, printing speed, relative density, and cell size, in the context of 3D-printed TMPS gyroid structures. The current study explored the optimized performance of gyroid TPMS structures using the Grey Relational Analysis (GRA). Gyroid TPMS structures hold promise as impact-absorbing materials for safeguarding delicate objects in packaging, enhancing protection in sports equipment, and even offering energy dissipation in blast protection panels.

## 2. Experimental Design and Methodology

In the first stage, this research required an extensive study of the experiment goals by conducting a literature review regarding the PLA specimen based on gyroid structures. Using the FDM technique with different printing speeds, gyroid samples were scheduled to be 3D printed and were eventually prepared to undergo mechanical testing in the form of a uniaxial compression test because a single test such as this provides a lot of useful information regarding a material’s behavior under compressive loading. Nine cuboid compressive specimens with gyroid-based structures were 3D printed using a TOBECA 3D printer (Tobeca, Vendôme, France). The specimens were initially designed using MS Lattice Software 1.0 [26], exported in STL format to the slicing software CURA 5.7.0, and then 3D printed. The specimens were printed in the flat orientation for this study. The 3D printer works on the principle of fused deposition modeling, wherein the melted thermal plastic continually flows through the nozzle and prints the specimen in the desired geometry.

The material used for printing the specimens was polylactic acid (PLA). A mechanical test like compressive testing was performed on the 3D-printed specimens, and the output results and data were analyzed. After benchmarking the PLA literature, the input parameters used for this study were printing speed, relative density, and unit cell size. Printing speed is the speed of the extruder depositing material in the XY plane. Relative density is a measure of how much material is filled in the printed volume. It is the size of a repeating building block in a structure that plays a significant role in strength and stiffness. The three chosen printing speeds were 22 mm/s, 33 mm/s, and 40 mm/s, which subsequently printed nine gyroid cube samples designed with different relative densities and unit cell sizes. To print the samples, the travel speed was selected to be 150 mm/s. The three selected relative densities were 20%, 40%, and 60%, whilst the three selected unit cell sizes were 1.58 mm, 3.17 mm, and 6.35 mm. 

**Figure 2:**
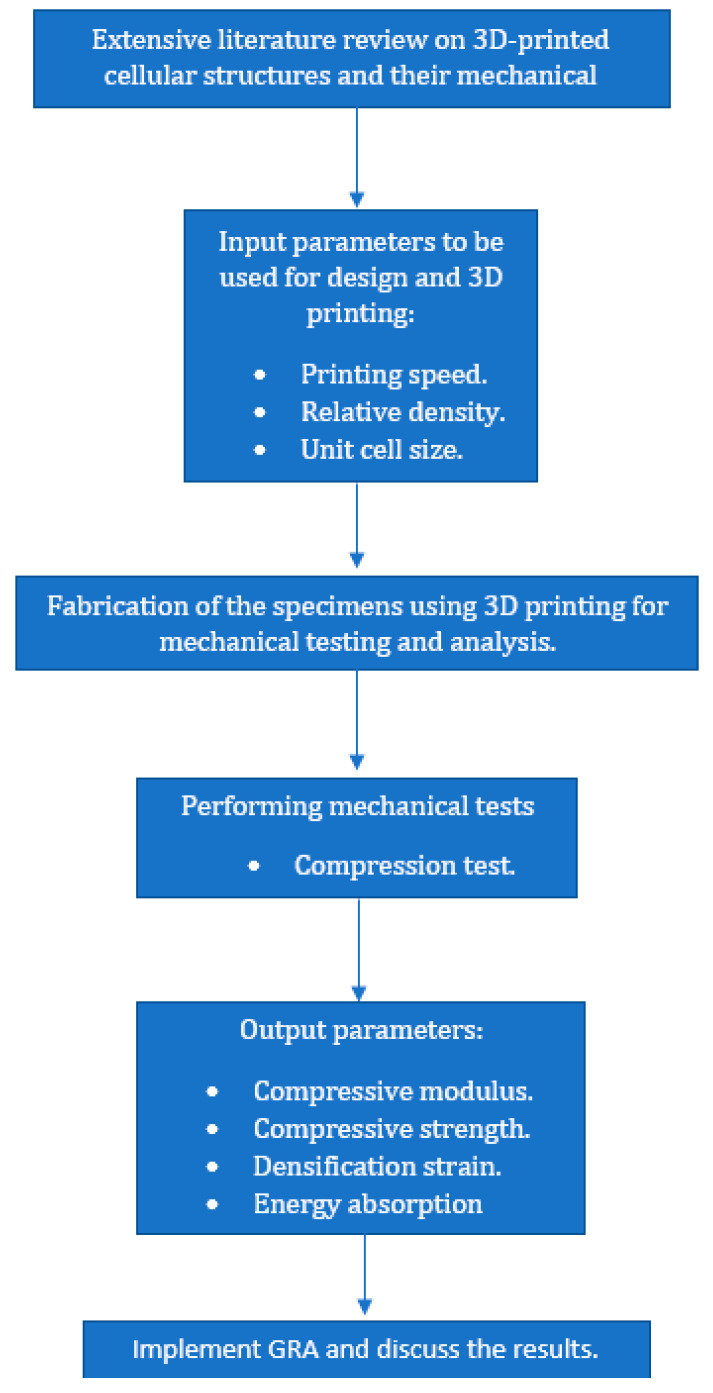
Methodology of this research study.

**Figure 3:**
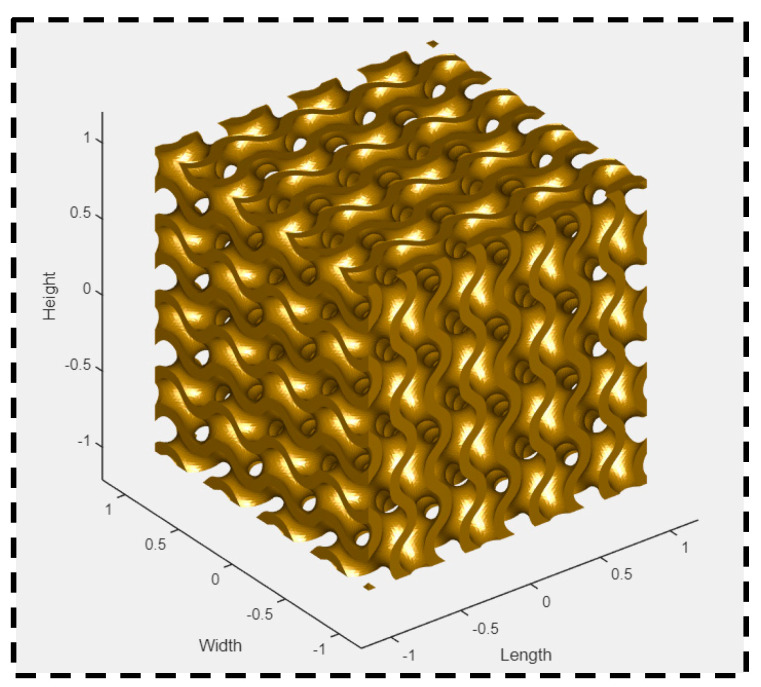
Using MS Lattice software to design to each specimen.

**Figure 4:**
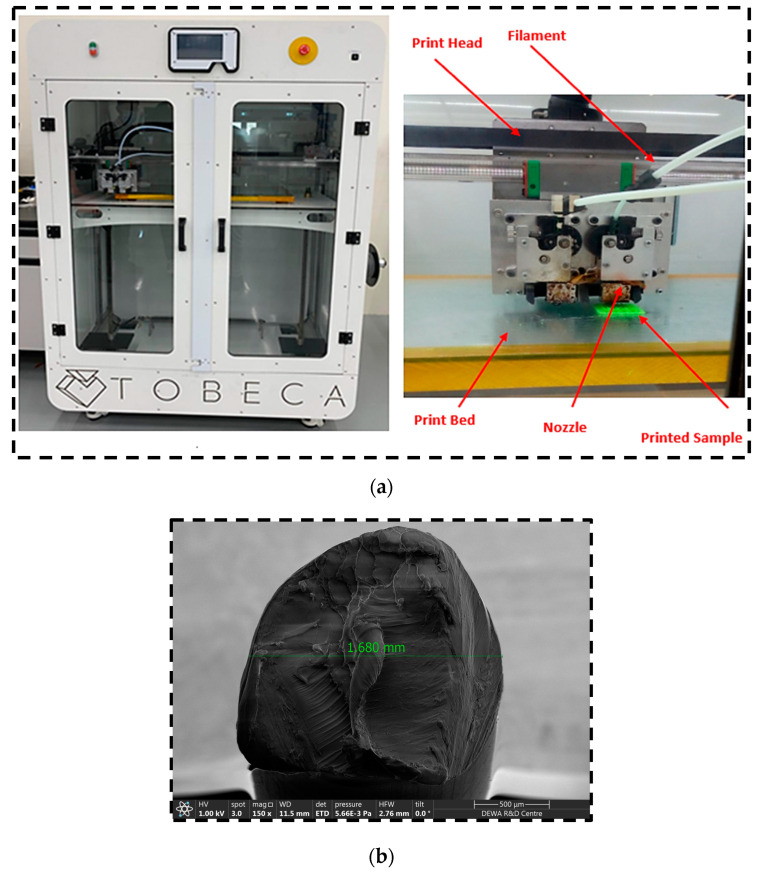
(**a**) TOBECA 3D printer used to fabricate the 3D-printed specimens. (**b**) Cross section of PLA filament.

The output parameters studied experimentally were ultimate compressive strength, the compressive modulus, and the toughness or energy absorbed. Figure 2 depicts the approach in which this research study was performed. Figure 3 shows the design of a cuboid gyroid sample in MS Lattice [32] to be saved in an STL file, which would then be sliced using CURA within Repetier Software 2.3.2. Figure 4 shows the TOBECA 3D printer used to print the specimens.

**Figure 5:**
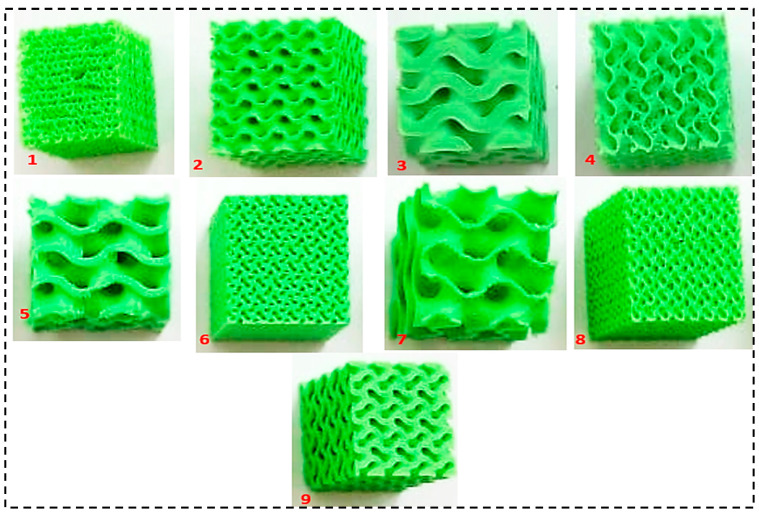
Three-dimensional-printed gyroid structures using an L09 Taguchi array.

**Figure 6:**
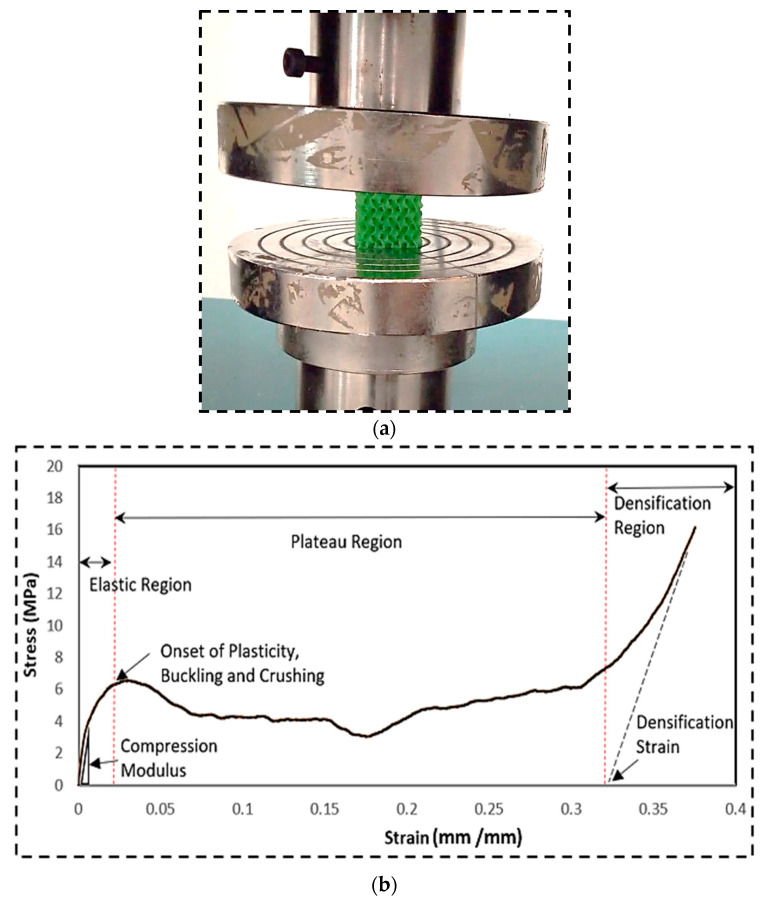
(**a**) Using the Universal Testing Machine to compress each 3D-printed specimen. (**b**) Stress–strain curve obtained during the run of Sample # 4 (printing speed = 33 mm/s, relative density = 20%, and cell size = 3.17 mm).

Figure 4a depicts the TOBECA 3D printer in the process of printing a gyroid sample. Figure 4b shows the cross-section of the PLA filament (3DTALK 3D printing filament Jiangsu Ouring 3D Technology Co., Ltd., Nantong, China, 1.75 mm PLA, recommended extrusion/nozzle temperatures: 190 °C to 220 °C). In Figure 5, a total of nine gyroid samples were printed using a Taguchi L09 orthogonal array, with two replications for each. Figure 6a shows the Universal Testing Machine (UTM) used to compress all the 3D-printed samples to study their mechanical properties. The PLA filament used had a diameter of 1.75 mm.

**Figure 7:**
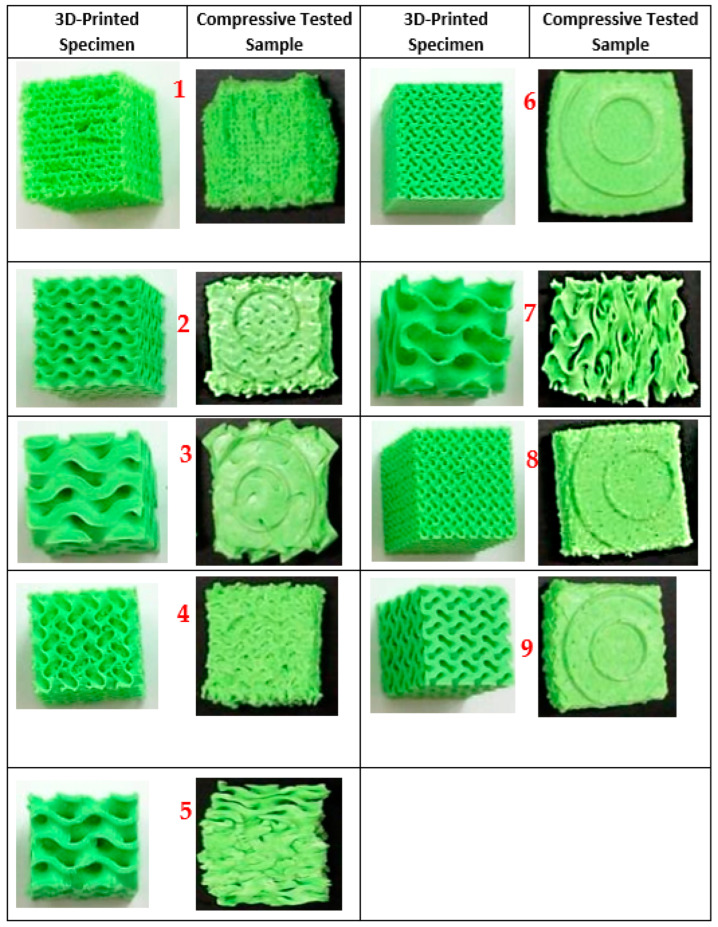
Compressed 3D-printed specimens.

Figure 7 shows the 3D-printed 09 samples after and prior to compressive testing. The compression modulus is an important parameter that indicates how well a material resists compressive loading. A higher value signifies a stiffer material. Densification strain is another important property. It describes how a porous material changes to a denser state under compressive loading. Toughness refers to the energy absorbed by a structure before it fractures. Plastic deformation and the buckling of cell walls can significantly contribute to a structure’s toughness. Yield strength provides information about the point at which plastic deformation begins in the material.

### Design of Experiment

The set of experiments performed in this study was modeled using the Taguchi design of experiments. The Taguchi orthogonal array of the L09 design used in this study consisted of an array table corresponding to three factors with nine runs. By using orthogonal arrays, the Taguchi method requires a significantly smaller number of experiments compared with full factorial designs, saving time and material resources. The Taguchi design was incorporated using Excel software version 2108. Table 2 shows the three input parameters used in the Taguchi design. To prepare, a compression test must be conducted on a solid specimen with the option selected as 100% infill density. This acts as a reference to all the other patterned specimens that are to be tested later. Parameters that were required to be output include the following: compressive modulus, compressive strength, and toughness. These three parameters were also set as the response variables. The independent variables in this study were printing speed, relative density, and unit cell size.

## 3. Grey Relational Analysis

**Figure 8:**
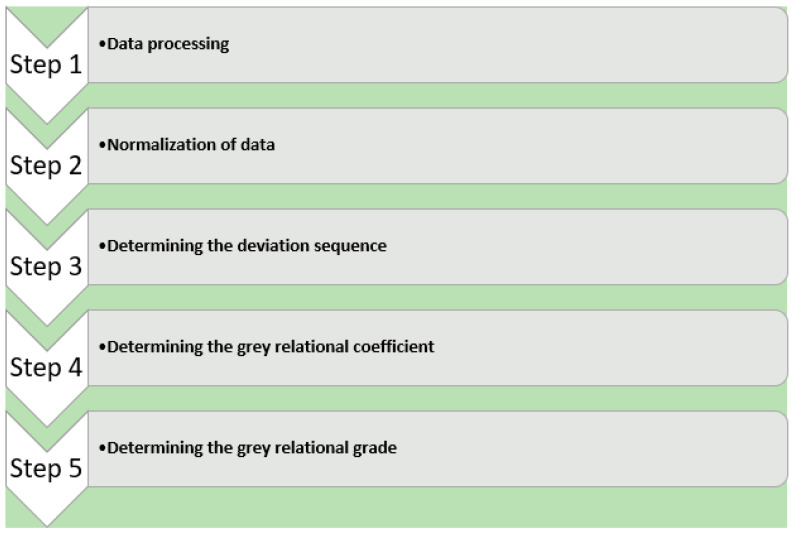
Multistep sequence used for grey relational analysis.

Grey relational analysis was implemented in this study to identify the best and most optimized combination of independent variables that yield the best mechanical properties. Excel software was used for grey relational analysis. Grey relational analysis was performed through a multiple-step sequence, as depicted below in Figure 8.

### 3.1. Phase 1—Data Processing

Uniaxial compressive tests were performed on the specimens, the output data were compiled, and calculations were performed to process the four output parameters, namely, yield strength, compression modulus, densification ratio, and energy absorption. The output data were processed in Excel. Table 3 shows the Taguchi output data. In Table 3, A, B, and C are the input parameters that represent printing speed, relative density, and unit cell size, respectively. The output parameters are yield strength, compression modulus, densification strain, and energy absorption, as shown in Table 3. The relative density has a stronger influence as can be seen in Table 3 for the first, fourth, and seventh runs. The lower value of yield strength can be attributed to the lower value (20%) of the relative density. 

### 3.2. Phase 2—Normalization of Data

The next phase in gray relational analysis is data normalization. Raw data can be normalized through a sequence of steps in Excel software. Three options are presented to choose from for normalizing the data. They are nominal, smaller, and larger is better. For this study, the larger is better choice was selected as all the mechanical properties studied were to be of the highest value for optimized and improved performance. 

For the larger is better option, a specific formula was used to normalize the data.

(1) $$x_{ij}=\frac{y_{ij}-\min\left(y_{ij}\right)}{\max\left(y_{ij}\right)-\min\left(y_{ij}\right)}\;$$

In the above formula, *y_ij_* represents the corresponding data points for the three output parameters (compressive modulus (Y), compressive strength (U), and toughness (E)) in Table 3, while *x_ij_* represents the resulting normalized data. The output data for the three studied parameters in Table 3 were normalized using Equation (1), and Table 4 displays the obtained results.

### 3.3. Phase 3—Determining the Deviation Sequence

The deviation sequence of the normalized data in Table 4 was obtained by normalizing the data points between the values of 0 and 1. The deviation sequence was calculated using Equation (2) as depicted below.

∆0*i* (*k*) = *x*0 (*k*) − *xi* (*k*) (2)

where ∆0*i* (*k*) represents deviation, *x*0 (*k*) represents reference, and *xi* (*k*) represents comparability. In this formula, the reference value was 1, while *xi* (*k*) was the set of normalized data points. The obtained deviation sequence responses are recorded in Table 5. The maximum deviation from the reference is 1, while the minimum deviation from the reference is 0.

### 3.4. Phase 4—Determining the Grey Relational Coefficient

The grey relational coefficient was calculated using Equation (3). The formula includes the data points from the deviation sequence responses.

(3) $$\varepsilon_{i}\;(k)=\frac{\Delta \min+\left(\psi \times \Delta max\right)}{\Delta ij+\left(\psi \times \Delta max\right)}$$

where Δmin is the minimum value of the deviation sequence, while Δmax represents the maximum value of the deviation sequence. Also, Δij represents the corresponding data points from the deviation sequence. For this study, the distinguishing coefficient ψ was set to 0.5. Δmin is 0, while Δmax is 1. Table 6 displays the grey relational coefficient (GRC) of the three output parameters.

### 3.5. Phase 5—Determining the Grey Relational Grade

The next step was to determine the grey relational grade (GRG). The GRG of each experiment was determined by computing the average of the response variables from the grey relational coefficient responses. Equation (4) shows the formula used to compute the grey relational grade.

(4) $$\gamma i=\frac{1}{n}\sum_{i=1}^{n}\left(k\right)$$

The variable *n* in the above equation represents the number of response variables. In this study, four response variables were studied, and thus, *n* = 4. The grey relational grades were then ranked and analyzed. Table 7 displays the grey relational grades along with their ranks. 

**Figure 9:**
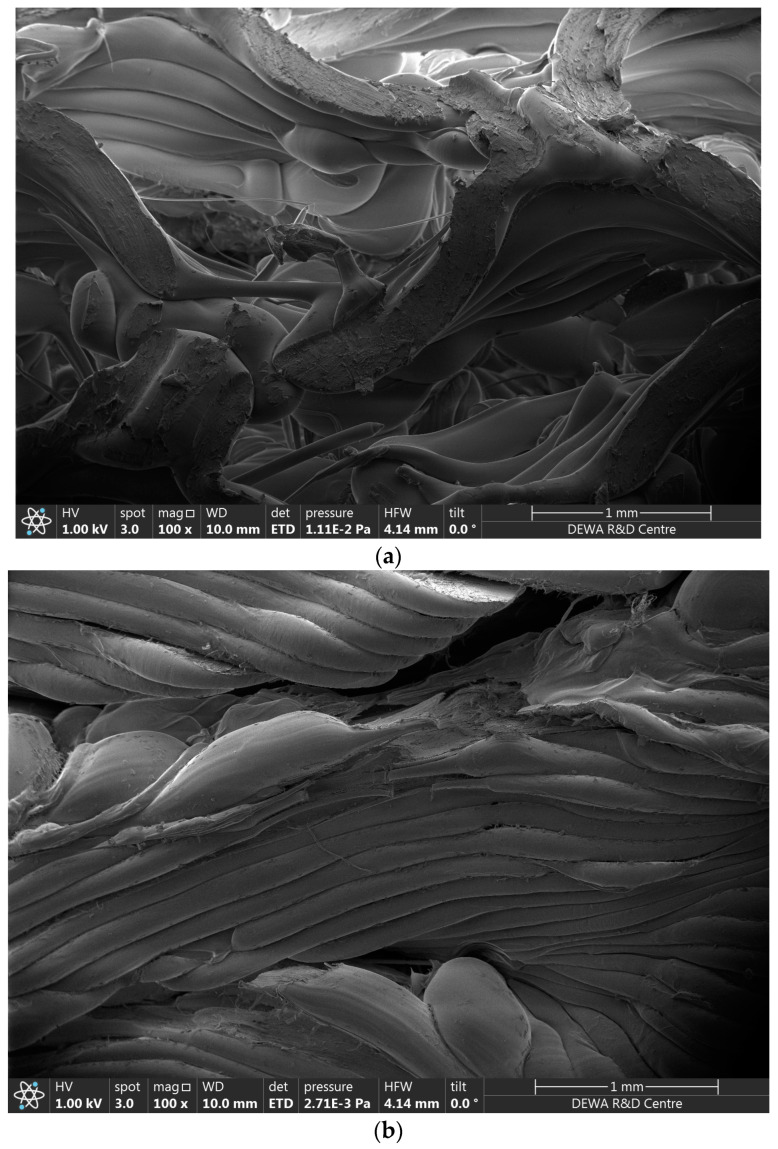
Scanning electron micrographs of (**a**) Sample # 1, ranked 9th, and (**b**) Sample # 9, ranked 1st.

The fracture against the best- and worst-ranked specimens was also investigated using scanning electron microscopy (SEM). The micrographs revealed the details of fracture in the samples. As per the literature, geometry, wall thickness, and stress distribution play a significant role in controlling fracture initiation and propagation. Complex cellular geometries with thin walls are more prone to stress concentrations and subsequent fracture. In the gyroid-based TPMS structure, sharp corners, junctions connecting cells and walls, and changes in strut thickness are the potential sites of stress concentration. Cracks may start and propagate from these locations. It has been witnessed that larger cell size has a tendency to lower stress concentration and increase fracture strength. The SEM micrograph of the first sample that ranked ninth is shown below in Figure 9. The sample showed very clearly broken walls and a fractured cellular structure without showing a lot of material flow. This highlights the brittle nature of the fracture facilitated by the stress concentration because of a lower cell size. The toughness value associated with this sample was also found to be the lowest among the other samples.

Figure 9b shows the scanning electron micrograph of the ninth sample that ranked first among the other samples. It can be clearly seen that print quality in the form of interlayer bonding was much better. The cell size was larger than the previously discussed sample, which resulted in a better distribution of stress and ended up lowering the stress concentration. The higher value of toughness in this sample clearly points out that the sample had less brittle and highly flexible behavior. 

**Figure 10:**
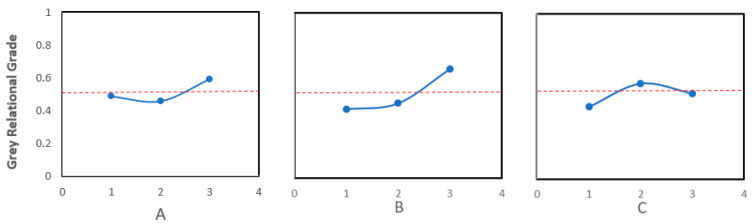
Plot of grey relational grade using the response table.

Table 8 indicates that Experiment 9 showed the highest grey relational grade. The input variable combinations in Experiment 9 displayed the most optimum conditions. The input variable combinations used in Experiment 9 were a printing speed of 40 mm/s, a relative density of 60%, and a unit cell size of 3.17 mm. Table 9 and Figure 10 provide the same information indicating that the optimal level was A3B3C2 in this work. Table 9 shows a comparison between the initial condition A1B1C1 and the optimal condition A3B3C2. It reveals that the grey relational grade improved by 0.2424.

## 4. Conclusions

In this study, the performance of gyroid-based TPMS structures was investigated using the variation in three input parameters, namely, cell size, sprint speed, and relative density. The TPMS gyroid structures were fabricated using material extrusion (MEx)-based 3D printing technology. Cuboid gyroid-based compressive specimens were modeled through CURA software and printed using a TOBECA 3D printer. The 3D-printed structures were exposed to a uniaxial compression test with a strain rate of 1 mm/min, and yield strength, compressive modulus, densification strain, and energy absorption were calculated as output parameters. In addition, the multi-objective optimization of grey relational analysis (GRA) was implemented. The following conclusions were drawn from this study.

- It was observed that a lower value (20%) of relative density provided the lowest value of yield strength. It can be concluded that relative density significantly controls the ability of the TPMS gyroid structure to resist plastic deformation.
- The ninth gyroid sample demonstrated the best yield strength, compressive modulus, and energy absorption. The ninth gyroid sample was printed with a printing speed of 40 mm/s, a relative density of 60%, and a cell size of 3.17 mm. The ninth experiment for the above-mentioned condition improved yield strength by 16.9%, the compression modulus by 34.8%, and energy absorption by 29.5% when compared with the second-best performer, the third experiment. The mechanism of toughness is linked with the plastic deformation and buckling of the cell wall. The optimal printing speed provides better material bonding because of the more precise alignment of layers. The optimal condition provided an improvement of 0.2424 in grey relational grade (GRG) when compared with the initial condition.
- To conclude, scanning electron micrographs were examined for the best- and worst-ranked samples. The micrographs confirmed the significant impact of geometry, wall thickness, and stress distribution on fracture behavior. The gyroid geometries generally involve thin walls, sharp corners, and thickness variations within structures that are regarded as potential sites of fracture development and crack formation due to stress concentrations. Larger cell sizes were observed to reduce stress concentration and enhance fracture strength. The SEM image of the first specimen (worst case), shows fractured walls and minimal material flow, exemplifying the brittle nature of the fracture arising from stress concentration because of its smaller cell size. This observation aligns with the lowest toughness value recorded for this specimen, further highlighting the critical role of design parameters in optimizing fracture resistance and mechanical performance in gyroid TPMS structures.

## Figures and Tables

**Table 1:** Default printer settings.

Printer Parameters	Set Values
Printing temperature	210 °C
Printer bed temperature	60 °C
Infill density	100%
Nozzle diameter	0.4 mm

**Table 2:** Taguchi design.

Run	A: Printing Speed (mm/s)	B: Relative Density (%)	C: Unit Cell Size (mm)	Weight (g)
1	22	20	1.58	5.7
2	22	40	3.17	12.6
3	22	60	6.35	19.7
4	33	20	3.17	5.86
5	33	40	6.35	13.13
6	33	60	1.58	19.2
7	40	20	6.35	6.07
8	40	40	1.58	11.59
9	40	60	3.17	19.9

**Table 3:** Output data array.

Run	A	B	C	Yield Strength (MPa)	Compression Modulus (MPa)	Densification Strain (%)	Energy Absorption (MJ/m^3^)
1	1	1	1	1.3	53.22	32.5	0.75
2	1	2	2	63.47	1999.6	37.5	24.34
3	1	3	3	122.89	4386.2	30.2	38.28
4	2	1	2	3.49	557.1	37.1	2.06
5	2	2	3	50	1500.7	36	11.46
6	2	3	1	52.1	4028.5	33	38.98
7	3	1	3	9.29	441.97	41	2.45
8	3	2	1	14.53	856	37.3	13.47
9	3	3	2	143.7	5913	30.8	49.58

**Table 4:** Normalized data.

Normalization				
Run	Yield Strength (MPa)	Compression Modulus (MPa)	Densification Strain (%)	Energy Absorption (MJ/m^3^)
1	0	0	0.2129	0
2	0.4365	0.3321	0.6759	0.4830
3	0.8538	0.7394	0	0.7685
4	0.0153	0.0859	0.6388	0.0267
5	0.3419	0.2470	0.5370	0.2192
6	0.3567	0.6784	0.2592	0.7829
7	0.0561	0.0663	1	0.0347
8	0.0929	0.1369	0.6574	0.2604
9	1	1	0.0555	1

**Table 5:** Deviation sequence.

Deviation Sequence				
Run	Yield Strength (MPa)	Compression Modulus (MPa)	Densification Strain (%)	Energy Absorption (MJ/m^3^)
1	1	1	0.7870	1
2	0.5634	0.6678	0.3240	0.5169
3	0.1461	0.2605	1	0.2314
4	0.9846	0.9140	0.3611	0.9732
5	0.6580	0.7529	0.4629	0.7807
6	0.6432	0.3215	0.7407	0.2170
7	0.9438	0.9336	0	0.9652
8	0.9070	0.8630	0.3425	0.7395
9	0	0	0.9444	0

**Table 6:** Grey relational coefficient.

Grey Relational Coefficient				
Run	Yield Strength (MPa)	Compression Modulus (MPa)	Densification Strain (%)	Energy Absorption (MJ/m^3^)
1	0.3333	0.3333	0.3884	0.3333
2	0.4701	0.4281	0.6067	0.4916
3	0.7738	0.6574	0.3333	0.6835
4	0.3367	0.3536	0.5806	0.3393
5	0.4317	0.3990	0.5192	0.3904
6	0.4373	0.6085	0.4029	0.6972
7	0.3462	0.3487	1	0.3412
8	0.3553	0.3668	0.5934	0.4033
9	1	1	0.3461	1

**Table 7:** Grey relational grades and rank.

Run	Grade	Rank
1	0.3471	9
2	0.4991	5
3	0.6120	2
4	0.4026	8
5	0.4351	6
6	0.5365	3
7	0.5090	4
8	0.4297	7
9	0.8365	1

**Table 8:** Response table for grey relational grades and ranks.

Response Table for Grey Relational Grade				
	1	2	3	Rank (Max–Min)
A	0.4861	0.4580	0.5917	0.1336 (3rd)
B	0.4196	0.4546	0.6617	0.2421 (1st)
C	0.4378	0.5794	0.5187	0.1416 (2nd)

**Table 9:** Confirmation test results.

	Levels	Yield Strength (MPa)	Compression Modulus (MPa)	Densification Strain (%)	Energy Absorption (MJ/m^3^)	Grey Relational Grade
Initial controllable parameters	A1B1C1	1.3	53.22	32.5	0.7536	0.3471
Optimal controllable parameters	A3B3C2					
Average experimental readings (02 replications)		128	2987.3	34.25	33.84	0.5895
Improvement in GRG = 0.2424						

## Data Availability

Data can be requested from the corresponding author due to privacy.

## Abbreviations

AM	additive manufacturing
CFRP	carbon fiber-reinforced polymer
CFRTPC	continuous fiber-reinforced thermoplastic composite
DLP	digital light processing
FDM	fused deposition Modeling
FFF	Fused Filament Fabrication
GRC	grey relational coefficient
GRG	grey relational grade
HUs	Hounsfield units
LPBF	laser powder bed fusion
MJF	Multi Jet Fusion
PACs	periodic auxetic cellular structures
PLA	polylactic acid
PBF	powder bed fusion
RCA	re-entrant chiral auxetic
SEM	scanning electron microscopy
STL	standard triangulation language
TPMS	Triply Periodical Minimal Surface
TCP/BG	tricalcium phosphate/bio glass composite
µCT	micro-computed tomography
